# Myelin Activates FAK/Akt/NF-κB Pathways and Provokes CR3-Dependent Inflammatory Response in Murine System

**DOI:** 10.1371/journal.pone.0009380

**Published:** 2010-02-23

**Authors:** Xin Sun, Xi Wang, Tianxiang Chen, Tianyi Li, Kai Cao, Andrew Lu, Yongxiong Chen, Dongming Sun, Jianhong Luo, Jianqing Fan, Wise Young, Yi Ren

**Affiliations:** 1 W. M. Keck Center for Collaborative Neuroscience, Department of Cell Biology and Neuroscience, Rutgers, The State University of New Jersey, Piscataway, New Jersey, United States of America; 2 Department of Neurobiology, Institute for Neuroscience, Zhejiang University School of Medicine, Hangzhou, People's Republic of China; 3 Statistics Laboratory, Princeton University, Princeton, New Jersey, United States of America; University of Milan-Bicocca, Italy

## Abstract

Inflammatory response following central nervous system (CNS) injury contributes to progressive neuropathology and reduction in functional recovery. Axons are sensitive to mechanical injury and toxic inflammatory mediators, which may lead to demyelination. Although it is well documented that degenerated myelin triggers undesirable inflammatory responses in autoimmune diseases such as multiple sclerosis (MS) and its animal model, experimental autoimmune encephalomyelitis (EAE), there has been very little study of the direct inflammatory consequences of damaged myelin in spinal cord injury (SCI), *i.e.,* there is no direct evidence to show that myelin debris from injured spinal cord can trigger undesirable inflammation *in vitro* and *in vivo*. Our data showed that myelin can initiate inflammatory responses *in vivo*, which is complement receptor 3 (CR3)-dependent via stimulating macrophages to express pro-inflammatory molecules and down-regulates expression of anti-inflammatory cytokines. Mechanism study revealed that myelin-increased cytokine expression is through activation of FAK/PI3K/Akt/NF-κB signaling pathways and CR3 contributes to myelin-induced PI3K/Akt/NF-κB activation and cytokine production. The myelin induced inflammatory response is myelin specific as sphingomyelin (the major lipid of myelin) and myelin basic protein (MBP, one of the major proteins of myelin) are not able to activate NF-κB signaling pathway. In conclusion, our results demonstrate a crucial role of myelin as an endogenous inflammatory stimulus that induces pro-inflammatory responses and suggest that blocking myelin-CR3 interaction and enhancing myelin debris clearance may be effective interventions for treating SCI.

## Introduction

Inflammatory response induced by trauma in the central nervous system (CNS), including traumatic brain injury (TBI) and spinal cord injury (SCI), contributes progressive neuropathology and reduction in functional recovery. The inflammatory response includes invasion of inflammatory cells, activation of CNS-resident glial cells and release of cytokines and chemokines [1], [2], [3]. Pro-inflammatory cytokines such as tumor necrosis factor-α (TNF-α), interleukin-1β (IL-1β), IL-6 and chemokines such as macrophage chemoattractant protein-1 (MCP-1), macrophage inflammatory protein-1α (MIP-1α), and chemokine (C-X-C motif) ligand 10 (CXCL 10), are produced by both infiltrating cells and CNS-resident glial cells, orchestrating a pathogenic cascade leading to inflammation and axonal damage [4]. TNF-α and IL-1 activate the nuclear factor kappa B (NF-κB) pathway and then further stimulate production of inflammatory mediators such as inducible nitric oxide synthase (iNOS) and prostaglandin E2 [5]. IL-6 not only mediates spinal cord neural injury via JAK/STAT activation [6] but may also be a potential inhibitor of neurogenesis [7]. In addition, these inflammatory processes enhance recruitment and activation of leukocytes, leading to amplification of inflammation and further tissue damage. However, there is no direct evidence to show where these pro-inflammatory cytokines come from and whether myelin is major stimulus responsible for inflammatory mediator production in SCI.

Myelin degeneration occurs after injury and in multiple sclerosis (MS) and its animal model, experimental autoimmune encephalomyelitis (EAE). Demyelinated areas in the CNS of patients with MS are characterized by inflammatory infiltrates that contain blood-derived myelin-specific T cells, B cells and macrophages. Degenerated myelin containing inhibitory molecules such as NogoA, Oligodendrocyte-myelin glycoprotein (OMgp) and myelin-associated glycoprotein (MAG) inhibits axon regeneration [8], [9] and further activates complement system to destroy intact myelin. Although it is well documented that degenerated myelin triggers undesirable inflammatory responses in MS and EAE, there has been very little study of the direct inflammatory consequences of damaged myelin in spinal cord injury (SCI), *i.e.* there is no direct evidence to show that myelin debris from injured spinal cord can trigger undesirable inflammation *in vitro* and *in vivo*. Thus, study on understanding the mechanisms underlying inflammatory reaction induced by myelin is crucial to prevent further neuronal damage and develop the anti-inflammatory treatments of SCI.

Our present results demonstrate that myelin debris contributes to inflammatory responses in animal models via stimulating cytokine production. We further show that myelin-increased cytokines expression is via activation of NF-κB through FAK/PI3K/Akt signaling pathway and complement receptor 3 (CR3)-dependent. Inhibiting NF-κB activation abrogates myelin-induced cytokine production on macrophages. Our study provides the first direct evidence that myelin-CR3 interaction triggers undesirable inflammation *in vitro* and *in vivo*.

## Methods

### Reagents and Antibodies

All chemicals were purchased from Sigma (St. Louis, MO) unless otherwise indicated. The BAY 11-7082 was obtained from Biomol (Plymouth Meeting, PA). The antibodies against FAK, phospho-FAK (Tyr-576/577), p85, phospho-p85 (Tyr-458), Akt, phospho-Akt (Ser-473), IκB-α, phospho-IκB-α (Ser-32/36) and β-actin were purchased from Cell Signaling Technology (Danvers, MA). The rabbit-anti-p65 antibody, goat-anti-MIF antibody and rabbit-anti-MIF antibody were obtained from Santa Cruz Biotechnology (Santa Cruz, CA). Rabbit-anti-IBA-1 antibody was from Wako USA (Richmond, VA). Rat-anti-Ly-6G (Gr-1) antibody was obtained from eBioscience (San Diego, CA). Alexa 546-conjugated goat-anti-rabbit IgG, FITC-conjugated rabbit-anti-rat IgG, HRP-conjugated goat-anti-rabbit IgG and HRP-conjugated rabbit-anti-mouse IgG antibodies were from Invitrogen (Carlsbad, CA).

### Cells and Mice

Bone marrows were harvested from C57BL/6 wild type (WT) and CR3 knockout (KO) mice (CD11b deleted) (The Jackson Laboratory, Bar Harbor, Maine) and cultured in DMEM containing 10% FBS and 15% L929 cells-conditioned medium as a source of M-CSF. Bone marrow-derived macrophages were used after 7–10 days of culture. All mice were maintained in pathogen-free animal facility in Rutgers University. Animal protocols were approved by Animal Care and Facilities Committee in Rutgers University.

### Myelin Preparation

Myelin was isolated from the brains of 3-month-old mice by sucrose density gradient centrifugation (0.32 M and 0.85 M) at 75000×g, as described previously [10]. The protein content of myelin was determined by Bradford method with bovine serum albumin as the standard. The endotoxin concentration of myelin debris was below the detection limit by Limulus Amebocyte Lysate assay. Myelin was used to stimulate cells with concentration of 100 µg myelin protein per milliliter in all experiments.

### Myelin Stimulation in Peritoneal Cavity

C57BL/6 WT and CR3 KO mice were injected *i.p.* with myelin (25 mg/kg body weight) or PBS alone for control. Mice were sacrificed at different time points and peritoneal lavage was performed. Cells in peritoneal lavage fluid were counted and stained with Diff-Quick staining kit (IMEB Inc., San Marcos, CA). Remaining lavage fluids were centrifuged at 400×g for 10 minutes and the cytokines in supernatants were detected by ELISA.

### Myelin Injection in Spinal Cord

Myelin in PBS was adjusted to 125 µg/kg body weight and a total volume of 0.5 µl was stereotaxically injected into the spinal cord at T8–T10 vertebrae by a T10 laminectomy using a microliter syringe (Hamilton Company, NV) fixed in a stereotaxi frame. Injection of PBS alone was used for control. Mice were sacrificed and perfused at 1, 4 and 7 days after injection, and spinal cords were fixed and frozen sectioned for immunostaining.

### Immunofluorescent Staining and Quantitative Analysis

Cells were seeded in 24-well plate at 1×10^5^ cells/well and incubated with culture medium containing myelin for different periods of time and then fixed in 4% paraformaldehyde (PFA) for 15 minutes at room temperature (RT). For the frozen section, spinal cord tissues were fixed in 4% PFA at 4°C overnight. After being washed with PBS for three times, cells or tissues were blocked in PBS with 1% BSA, 0.4% Triton X-100 for 20 minutes, then incubated with primary antibody for at 4°C overnight. After the incubation with secondary antibody for 1 hour, all images were acquired at RT using Zeiss Axiovert 200 M Microscope (Carl Zeiss, Germany) with 20×/0.4, equipped with AxioCam camera (Carl Zeiss, Germany) and software Axiovision 4.6 (Carl Zeiss, Germany). Injection regions and marginal regions were evaluated, six fields within each region were randomly selected. The numbers of Ly-6G and IBA1 positive cells were counted and divided by areas for each field (pixel×pixel). Numbers of cells per 100,000 pixel×pixel area were obtained as cell numbers per arbitrary unit for further analysis.

### Western Blot

Cells were washed with PBS, and directly lysed in RIPA buffer containing phosphatase inhibitors and proteinase inhibitors. Protein samples were subjected to SDS-polyacrylamide gel electrophoresis (SDS-PAGE). Proteins on the gel were transferred onto nitrocellulose membranes (GE Healthcare, UK) that were blocked with 5% milk in Tris-Buffered Saline containing 0.1% Tween 20 (TBST) for 1 hour at RT. Afterwards, the membranes were incubated with the indicated primary antibodies overnight at 4°C. After being washed with TBST, the membranes were incubated with the appropriate secondary antibodies. The immunoreactive bands were detected by using ECL Plus Western Blotting Detection System (GE Healthcare, UK) and analyzed by Kodak Molecular Imaging Software (v4.0.4) for intensities. Phosphorylation levels were shown by normalizing intensities of phosphorylated proteins to those of non-phosphorylated total proteins.

### RNA Isolation and Quantitative Real-Time (qRT)-PCR

Cells were incubated with myelin for 6 and 12 hours with or without the pre-incubation of inhibitors. Total RNA was isolated by TRIZOL method under the instruction of user manual and reverse-transcribed into cDNA by using oligo-dT primers and SuperScript II reverse transcriptase (Invitrogen). The primers used were shown in Supporting Information Table S1. The ABI 7900HT detection system (Applied Biosystems, UK) was used for qRT-PCR. SYBR Green dye (Applied Biosystems, UK) was used to monitor the replication of PCR products. Quantities of products were obtained by standard curve and then normalized to GAPDH quantity. The gene expression level was represented by the ratio of gene/GAPDH.

### Enzyme-Linked Immunosorbent Assay (ELISA)

The supernatants of cells incubated with myelin were collected for detection of mouse TNF-α, IL-1β and MIF. ELISA for mouse IL-1β and TNF-α were performed by Duoset ELISA Development kit from R&D Systems (Minneapolis, MN). ELISA for mouse MIF was done under the instruction of manual for human MIF ELISA kit from R&D Systems.

### Statistical Analysis

Data in figures are presented as mean ± SEM, with *n* representing the number of experiments. Statistical significance was evaluated using Student's unpaired *t*-tests or one-way analysis of variance (ANOVA) followed by Dunnett's post-test for multiple comparisons among groups. Statistical significance was set at *P* value <0.05.

## Results

### Myelin Is a Direct Inflammatory Stimulus which Induces Inflammation in Animal Models

**Figure 1:**
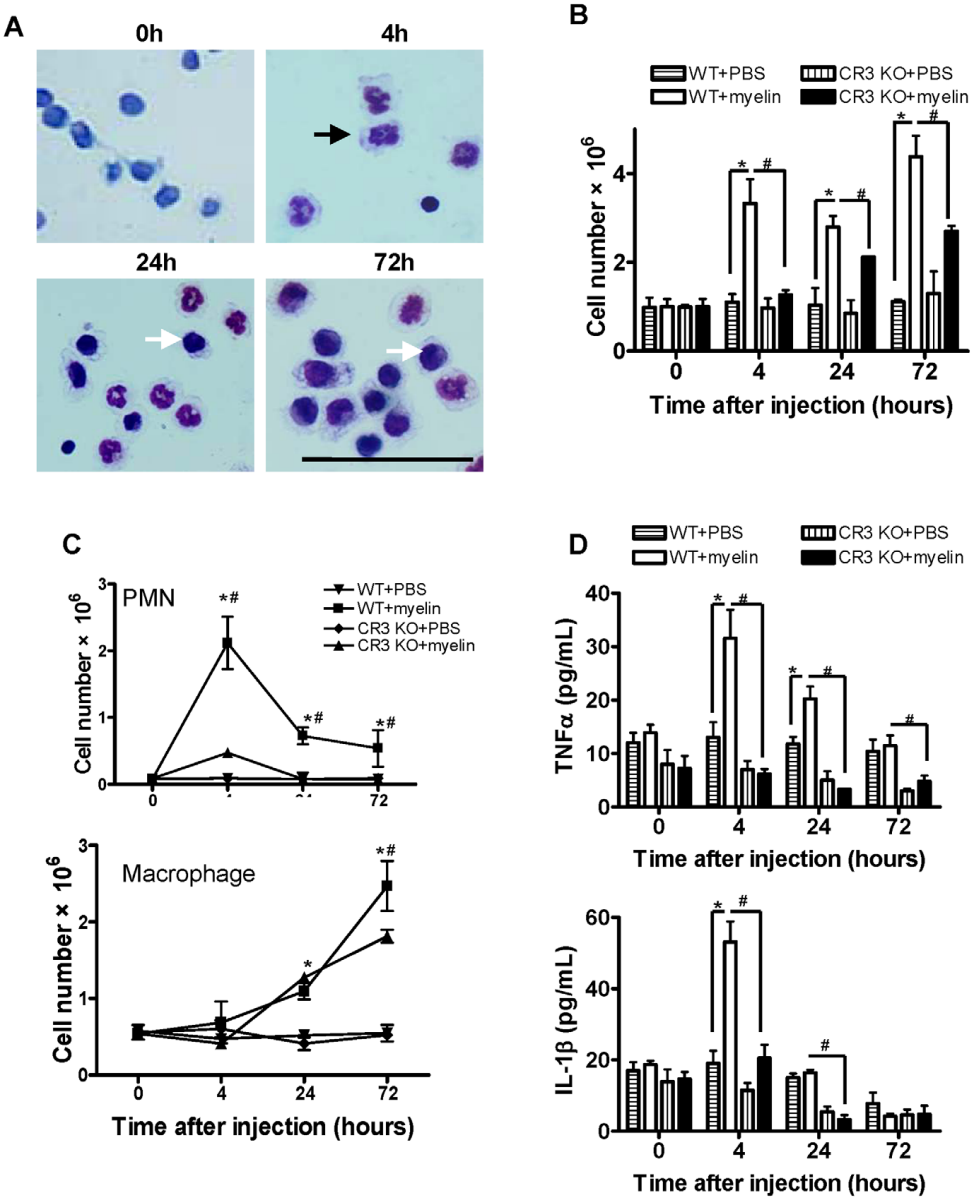
Inflammatory effect of myelin in peritoneal cavity from WT and CR3 KO mice. (A) Morphology of infiltrated cells in peritoneal cavity from WT mice. Neutrophils were indicated with black arrow and macrophages were indicated with white arrow. (Scale bar = 50 µm). (B) The total number of cells in peritoneal cavity. (C) Number of infiltrated neutrophils and macrophages in peritoneal cavity. (D) Levels of TNF-α and IL-1β in peritoneal lavage fluid detected by ELISA. Data were expressed as mean ± SEM. (n = 4 at each time point, *P<0.05 vs. WT + PBS; ^#^P<0.05 vs. CR3 KO + Myelin; *t*-test).

Although it is well documented that degenerated myelin triggers undesirable inflammatory responses in MS and EAE, there has been very little study to show that myelin debris from injured spinal cord can trigger undesirable inflammation directly *in vitro* and *in vivo*. We first assessed whether myelin is an inflammatory stimulus *in vivo*. We used mouse peritonitis model, a well-documented experimental animal model for studying pathogenesis of inflammatory stimuli and evaluating *in vivo* efficacy of anti-inflammatory and anti-infectious treatments. We administrated myelin (25 mg/kg body weight) or PBS (as control) intraperitoneally (*i.p.*) to C57BL/6 WT and CR3 KO mice, respectively and then performed peritoneal lavage and collected cells at 0, 4, 24 and 72 hours after the myelin injection. The number of cells in peritoneal lavage rapidly increased after myelin injection in WT mice (Figure 1A) and few neutrophils were found in mice injected with PBS alone. The total number of cells in WT mice at all time points was significantly higher than that of CR3 KO mice (Figure 1B). Furthermore, neutrophil infiltration dramatically increased in WT mice by 4 h after myelin injection and then declined quickly at 24 h after injection (Figure 1C). In comparison, neutrophil infiltration in CR3 KO mice was significantly reduced at all time points (Figure 1C). Macrophage infiltration started at 72 h after myelin injection in WT mice (Figure 1C) and the CR3 KO mice showed the less macrophage infiltration compared to WT mice (Figure 1C). In addition, increased levels of IL-1β and TNF-α in peritoneal lavage fluid were detected at time 4 h and declined after 24 h (Figure 1D) and significantly higher levels of IL-1β and TNF-α were detected in WT mice compared to CR3 KO mice (Figure 1D). By contrast, peritoneal injection of PBS did not induce significant cell infiltration and cytokine production in both WT and CR3 KO mice (Figure 1).

**Figure 2:**
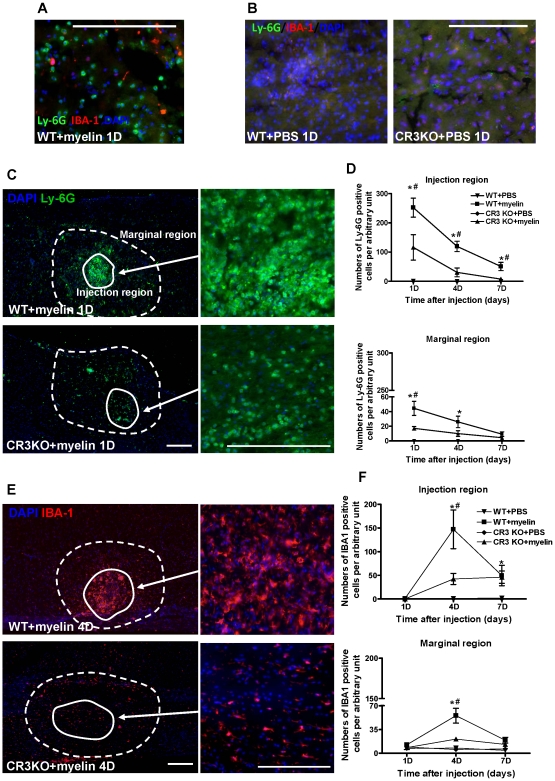
Inflammatory effect of myelin in spinal cord from WT and CR3 KO mice. (A) Neutrophils were positive for Ly-6G (green) but negative for IBA-1 (red) detected by double immunostaining. (B) Neutrophil and macrophage infiltration in spinal cord at 1 d after PBS injection detected by double immunostaining. (C) Neutrophil infiltration (Ly-6G^+^, green) in spinal cord at 1 d after myelin injection, along with injection region (solid line), marginal region (dashed line) and enlarged injection region. (D) The number of neutrophils at injection region and marginal region in spinal cord at 1 d after myelin or PBS injection. (E) Macrophage infiltration (IBA-1^+^, red) in spinal cord at 4 d after myelin injection, along with injection region (solid line), marginal region (dashed line) and enlarged injection region. (F) The number of macrophages at injection and marginal region in spinal cord at 4 d after myelin or PBS injection. Scale bar = 200 µm. Data were expressed as mean ± SEM. (n = 4 at each time point, *P<0.05 vs. WT + PBS; ^#^P<0.05 vs. CR3 KO + myelin; *t*-test).

We further assessed whether myelin is able to generate inflammatory response in mouse spinal cord. Myelin at total volume of 0.5 µL (125 µg/kg body weight) or PBS was stereotaxically injected into the spinal cord at T8–T10 vertebrae by a T10 laminectomy in WT and CR3 KO mice. Mice were sacrificed at 1, 4 and 7 days after myelin injection. Myelin injection induced large amount of leukocyte infiltration (Figure 2). We assessed the infiltration of macrophages and neutrophils by using IBA-1 (ionized calcium-binding adaptor molecule 1) and Ly-6G (lymphocyte antigen 6 complex, locus G, also known as Gr-1), respectively. The mouse Gr-1 antigen is a G-protein linked meyloid differentiation marker also known as Ly-6G [11]. Double immunostaining showed that infiltrated neutrophils were Ly-6G positive but negative for IBA-1 (Figure 2A). Kinetics of these cells infiltrated into the spinal cord is much earlier than IBA-1 positive macrophages (Figure 2C, D, E and F). Injection of PBS did not induce significant neutrophil infiltration in either WT or CR3 KO mice (Figure 2B and D). In myelin injection region in the spinal cord from CR3 KO mice, neutrophil infiltration was significantly reduced by more than 50% relative to that in WT mice at all time points after myelin injection (Figure 2C and D). Similar pattern can be observed in the marginal region of myelin injection (Figure 2C and D). After 7 days of myelin injection, few neutrophils can be observed in CR3 KO mice, while neutrophils in the spinal cord from WT mice still can be detected (Figure 2D). In addition, macrophages were not detected within the injection region in either WT or CR3 KO mice at 1 day after myelin injection. The number of macrophages significantly increased by 4 days at injection and marginal region in WT mice (Figure 2E and F). Macrophage infiltration was significantly suppressed in CR3 KO mice at 4 days after myelin injection (Figure 2E and F). However, there were no significant differences for macrophage infiltration between WT and CR3 KO mice at 7 days post-injection (Figure 2F). By contrast, injection of PBS did not induce significant macrophage infiltration in either WT or CR3 KO mice (Figure 2B and F).

### Effect of Myelin on Pro-Inflammatory Cytokine Expression *In Vitro*

**Figure 3:**
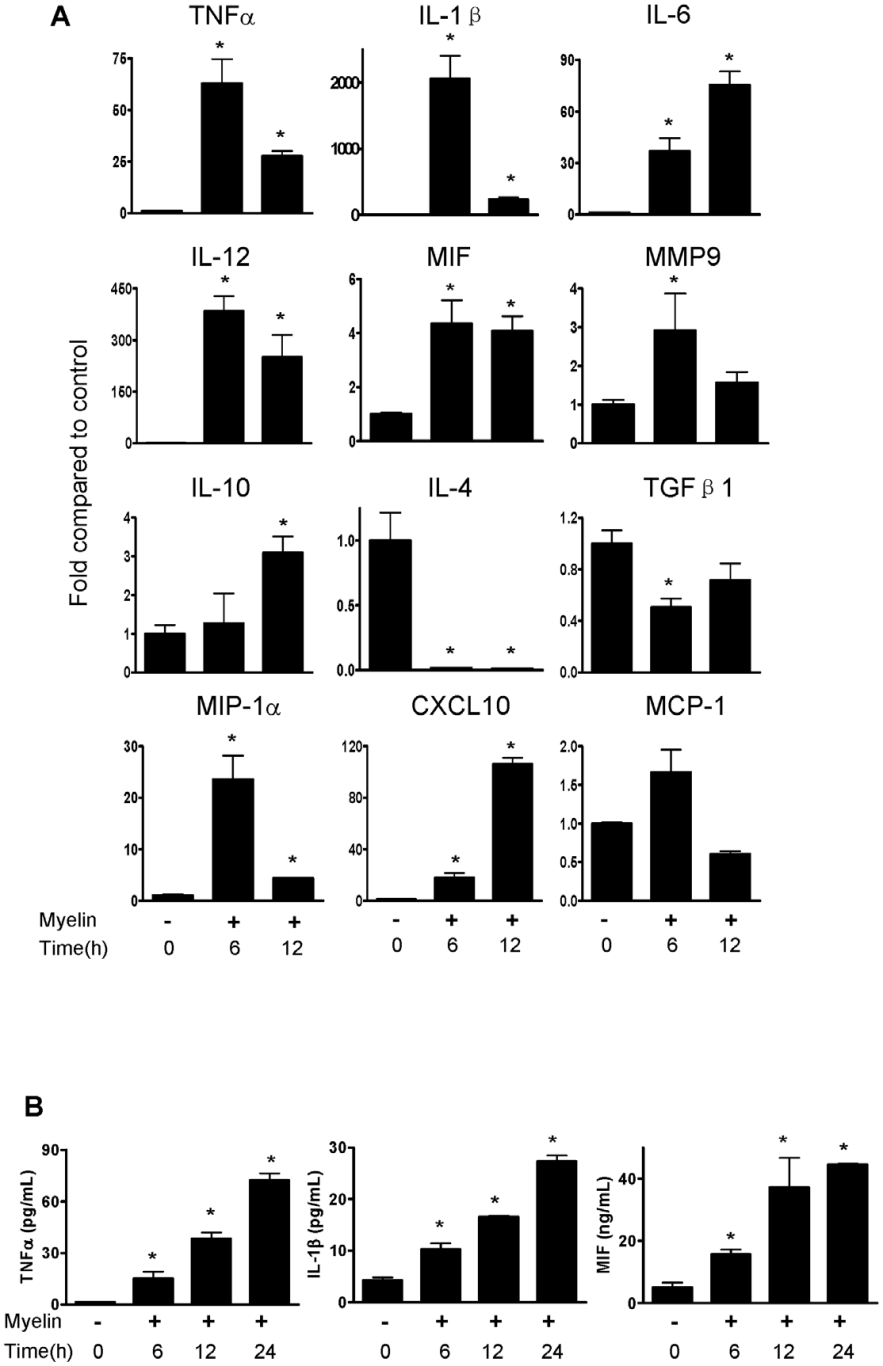
Expression of inflammatory mediators at transcriptional and translational levels in bone marrow-derived macrophages regulated by myelin. (A) Cytokines and chemokines in the cells detected by qRT-PCR. (B) Cytokines in the supernatants of cell culture detected by ELISA. Data were expressed as mean ± SEM and similar results were obtained from three independent experiments. (n = 3. *P<0.05 vs. control; one way ANOVA with Dunnett's post-test).

We then determined whether myelin treatment has effects on pro-inflammatory cytokine expression by inflammatory cells such as macrophages. We performed quantitative real-time (qRT)-PCR and ELISA on mouse bone marrow-derived macrophages to measure mRNA and protein levels of cytokines after myelin stimulation. As shown in Figure 3, mRNA levels of several of cytokines increased at 6 h and 12 h after myelin treatment. There was dramatic increase in expression of TNF-α, IL-1β, IL-6 and IL-12. Macrophage migration inhibitory factor (MIF) and matrix metalloproteinase 9 (MMP9) were also increased by myelin stimulation (Figure 3A). Interestingly, the expression of anti-inflammatory cytokines such as IL-4 and TGFβ1 was inhibited by myelin (Figure 3A). In addition to pro-inflammatory cytokines (*e.g.* TNF-α and IL-1β), expression of chemokines MIP-1α/CCL3 (chemokine (C-C motif) ligand 3) and CXCL10/IP-10 (interferon-inducible protein 10) significantly increased in macrophages stimulated by myelin (Figure 3A). However, myelin did not significantly enhance MCP-1/CCL2 production (Figure 3A). The protein levels of TNF-α, IL-1β and MIF secreted by macrophages treated with myelin were confirmed by ELISA (Figure 3B). Myelin exerts a time-dependent effect on expression of TNF-α, IL-1β and MIF.

### Myelin Induces NF-κB Activation

**Figure 4:**
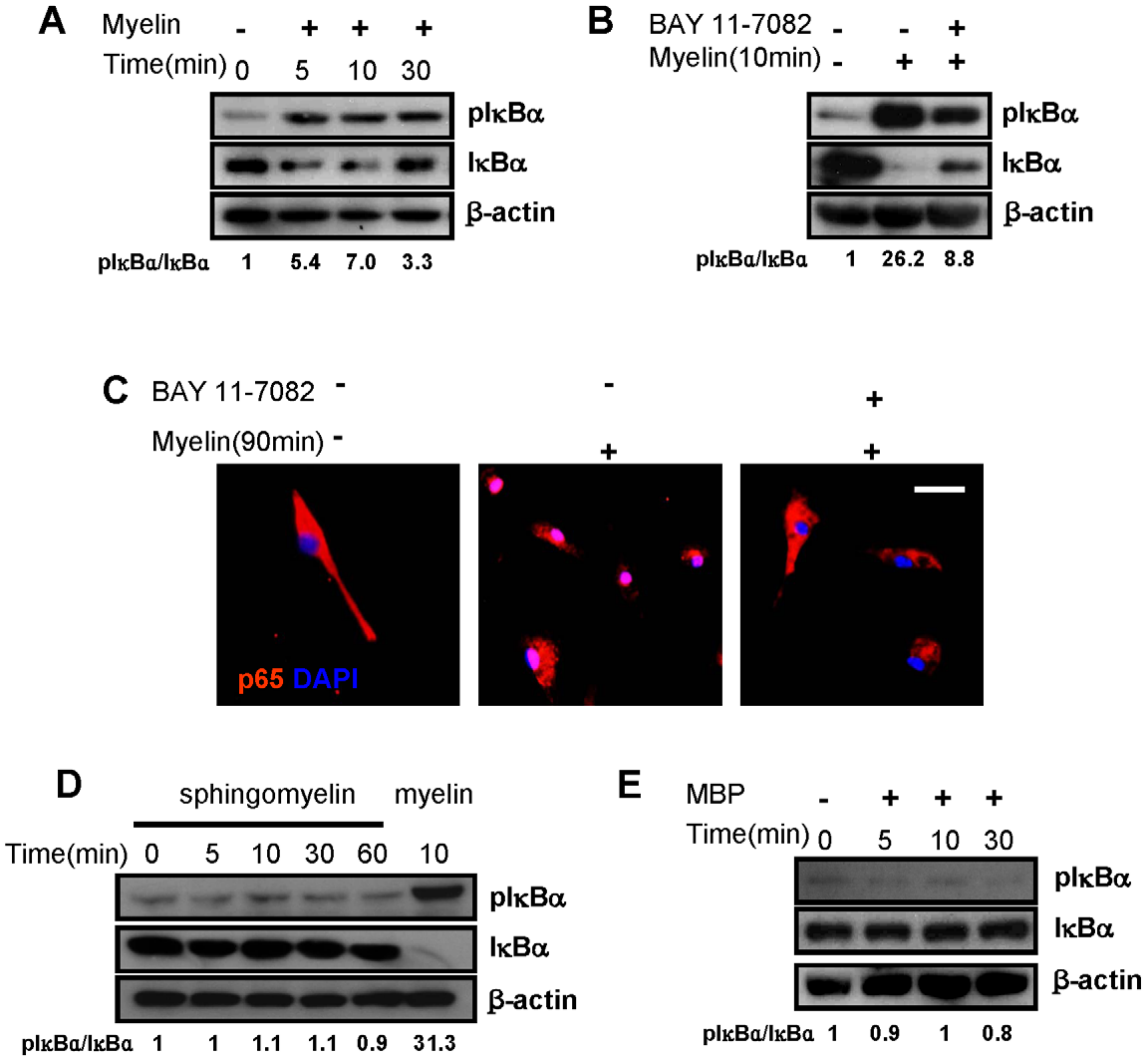
Effect of myelin on NF-κB activation. (A) Bone marrow-derived macrophages were incubated with myelin for indicated time and phosphorylation/degradation of IκB-α were assessed by western blot. (B) Effect of NF-κB inhibitor BAY 11-7082 on myelin-induced IκB-α phosphorylation and degradation. (C) Myelin-induced p65 translocation in macrophages detected by immunofluorescent staining. p65 was stained red and nuclei were stained with DAPI (blue) (Scale bar = 10 µm). (D) Sphingomyelin (25 µg/mL) had no effect on IκB-α phosphorylation and degradation macrophages. (E) MBP (1 µg/mL) had no effect on IκB-α phosphorylation and degradation in bone marrow-derived macrophages. The inhibitor was used for 30-minute pre-incubation before myelin incubation. Phosphorylation levels were normalized to non-phosphorylated total proteins. Similar results were obtained from three independent experiments.

We investigated signaling pathways triggered by myelin in macrophages. NF-κB is a key participant centrally in the inflammatory response to lipopolysaccharide (LPS) and other pro-inflammatory stimuli. We therefore examined the effects of NF-κB modulation on myelin-induced stimulation of macrophages. Myelin treatment increased the level of phosphorylated IκB-α, an inhibitory subunit of NF-κB, from 5 minutes up to 30 minutes (Figure 4A). Meanwhile, there was degradation of total IκB-α after myelin treatment (Figure 4A). Phosphorylation of IκB-α is considered to induce its degradation, which then releases NF-κB and confers it transcriptional activity. Pre-incubation with NF-κB inhibitor BAY 11-7082 (10 µM) for 30 minutes blocked IκB-α activation induced by myelin (Figure 4B). We also did immunofluorescence to detect subcellular distribution of p65, a major component of NF-κB. As shown in Figure 4C, p65 (stained as red) was located in the cytoplasm in the absence of myelin. After 90 minutes of exposure to myelin, p65 moved into the nuclei and this translocation can be abolished by pre-incubation of BAY 11-7082 (Figure 4C). The data indicate that myelin rapidly activates NF-κB in macrophages. By contrast, sphingomyelin (25 µg/mL), the major lipid component of myelin and MBP (1 µg/mL) are not able to activate NF-κB pathways (Figure 4D and E).

### Inhibition of NF-κB Activity Suppresses Myelin-Induced Cytokine Expression

**Figure 5:**
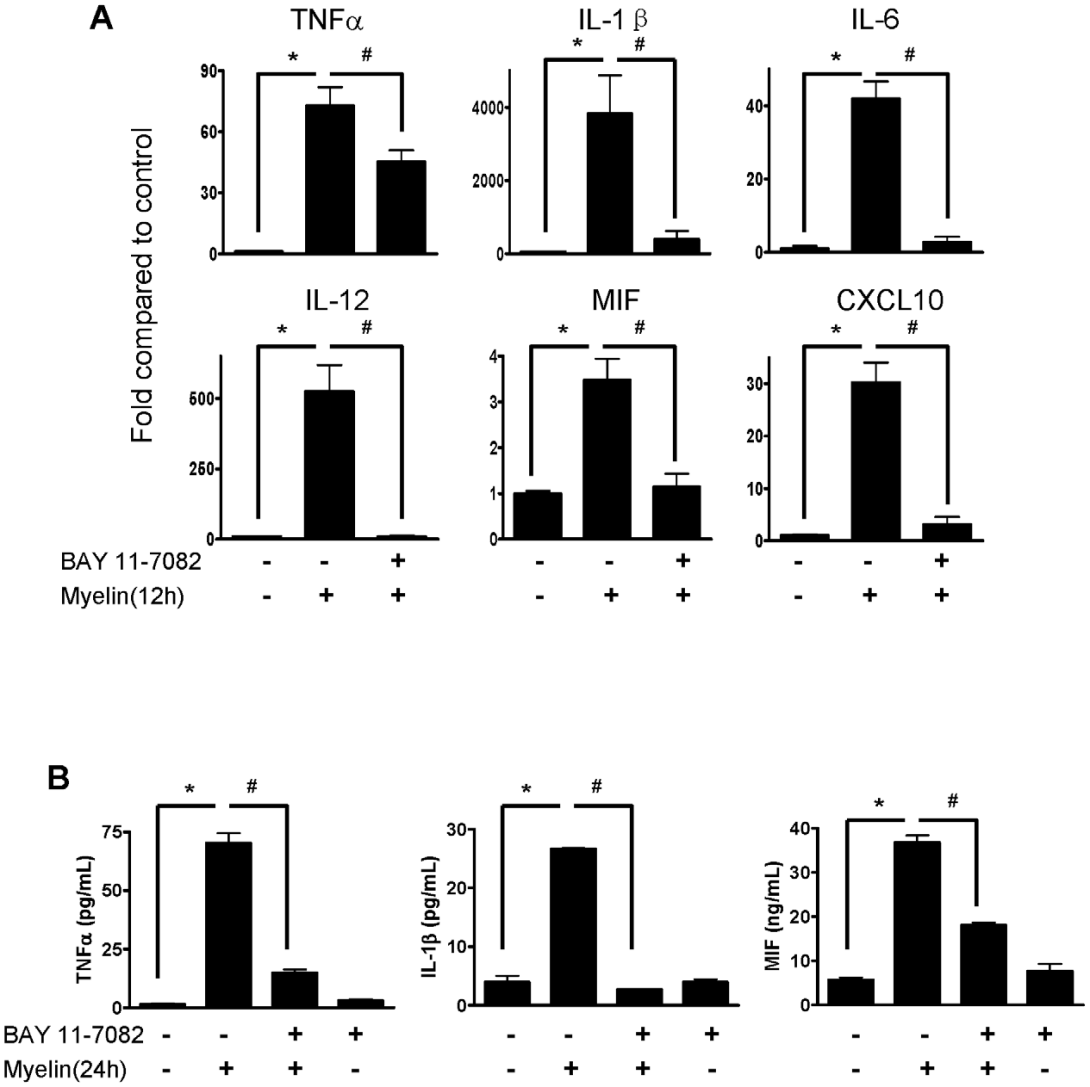
Effect of inactivation of NF-κB on myelin-induced cytokine expression in macrophages. (A) Macrophages were pre-treated with BAY 11-7082 for 30 minutes and then treated with myelin for 12 hours. Cytokines at transcriptional level were detected by qRT-PCR. (B) Macrophages were pre-treated with BAY 11-7082 for 30 minutes and then treated with myelin for 24 hours. Cytokines at protein level were detected by ELISA. Data were expressed as mean ± SEM and similar results were obtained from three independent experiments. (n = 3, *P<0.05 vs. control; ^#^P<0.05 vs. BAY treatment; *t*-test).

We further investigated whether inhibition of NF-κB activity could suppress the myelin-induced cytokine expression. Macrophages were pre-incubated with BAY 11-7082 for 30 minutes and then incubated with myelin for 12 or 24 hours. BAY 11-7082 significantly inhibited myelin-induced expression of TNF-α, IL-1β, IL-6, IL-12, MIF and CXCL10 at mRNA level (Figure 5A) detected by qRT-PCR and the protein level by ELISA (Figure 5B). These data indicate that myelin is a strong pro-inflammatory stimulus which can enhance the expression of a variety of pro-inflammatory cytokines and chemokines in macrophages by activation of NF-κB. Inhibition of NF-κB activation abrogates the stimulatory effects of myelin on cytokines production by macrophages.

### Myelin-Induced NF-κB Activation and Cytokine Expression Is CR3-Dependent

**Figure 6:**
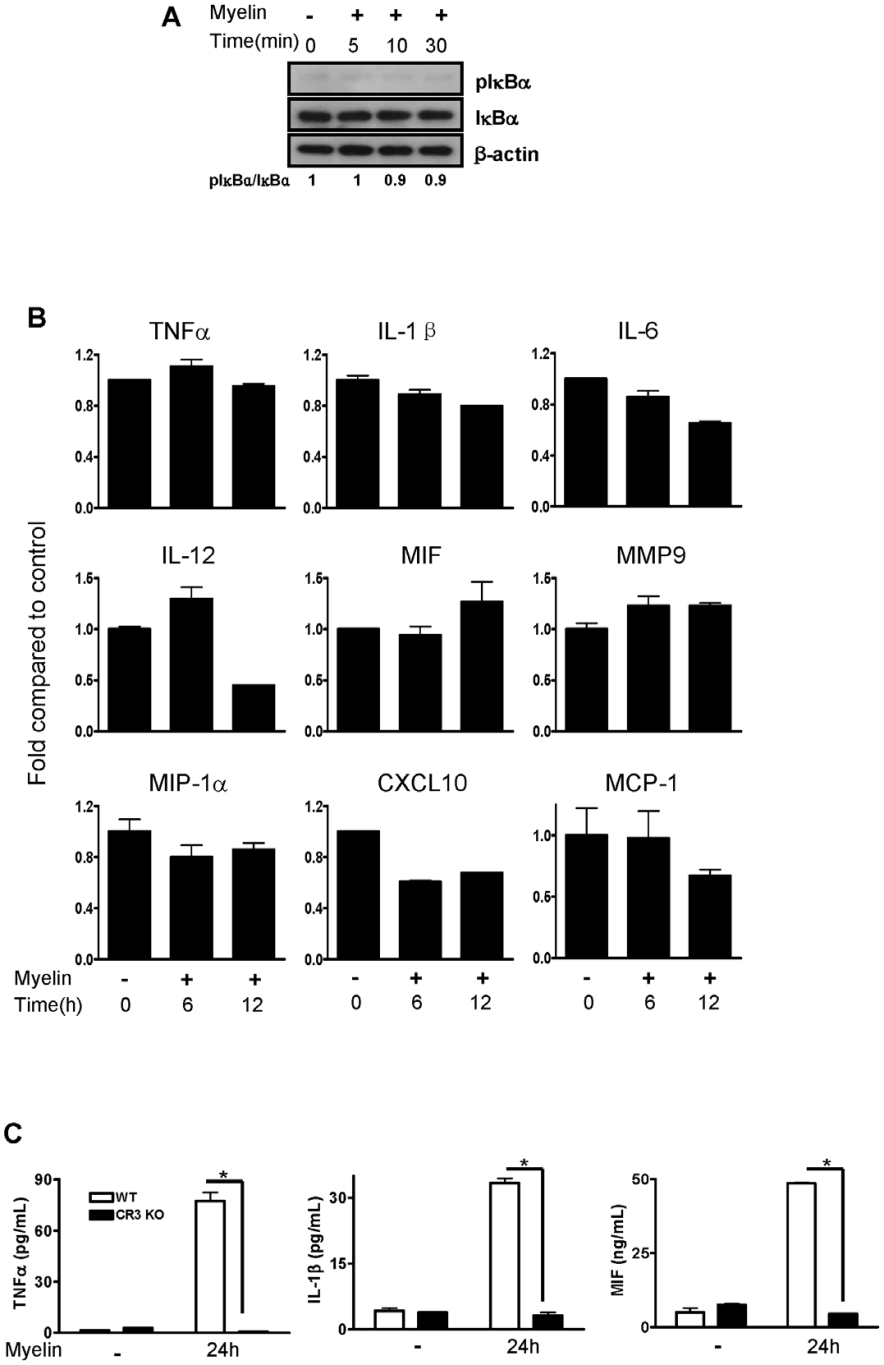
Effect of myelin on NF-κB activation and cytokine expression in bone marrow-derived macrophages from CR3 KO mice. (A) Macrophages from CR3 KO mice were treated with myelin for the indicated time and phosphorylation/degradation of IκB-α were assessed by western blot. (B) Macrophages from CR3 KO mice were treated with myelin for 6 and 12 hours. Cytokine and chemokine expression in cells at mRNA levels were detected by qRT-PCR. (C) Macrophages from WT and CR3 KO mice were treated with myelin for 24 hours and cytokines in the supernatants of cell culture were detected by ELISA. Phosphorylation levels were normalized to non-phosphorylated total proteins. Data were expressed as mean ± SEM and similar results were obtained from three independent experiments. (n = 3, *P<0.05 vs. CR3 KO + myelin; *t*-test).

Binding and phagocytosis of myelin by macrophages is mediated by complement receptor 3 (CR3, CD11b/CD18), a member of integrin family [12], [13]. CR3 interacts with many molecules in the extracellular matrix (ECM), such as iC3b and pathogens. Binding of ECM (*e.g.* fibrinogen, collagen, ICAM-1, and ICAM-2) with integrin, including CR3/MAC-1, can induce integrin activation and then regulate a variety of cell functions, including proliferation, apoptosis, migration and survival. We therefore ascertained whether myelin-induced NF-κB activation and cytokine expression were CR3-dependent. Myelin cannot phosphorylate and degrade IκB-α in bone marrow-derived macrophages from CR3 KO mice (Figure 6A). Furthermore, macrophages from CR3 KO mice did not respond to myelin by increasing mRNA (Figure 6B) and protein expression (Figure 6C) of cytokines.

### Myelin Induces FAK Phosphorylation

**Figure 7:**
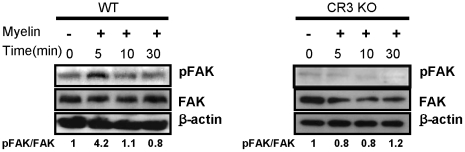
Effect of myelin on FAK phosphorylation. Bone marrow-derived macrophages from WT and CR3 KO mice were treated with myelin for the indicated time and phosphorylation of FAK (pFAK) was assessed by western blot. Phosphorylation levels were normalized to non-phosphorylated total proteins. Similar results were obtained from three independent experiments.

Next we studied to reveal the upstream molecules involved in myelin-induced NF-κB activation. Myelin interacts with CD11b subunit of CR3 and the ligand-CR3 interaction regulates a variety of biological functions through activation of its downstream effector, focal adhesion kinase (FAK) [14], [15]. FAK is a protein tyrosine kinase that is important in serving integrin signaling pathways [16]. Activated FAK interacts with Src family kinases or PI3K to initiate downstream signaling pathways [17]. We therefore determined whether NF-κB activation by myelin is via FAK activation. Figure 7 shows that FAK underwent transient FAK^Tyr-576/577^ phosphorylation at 5 minutes after myelin treatment in macrophages. The phosphorylation of FAK (pFAK) by myelin was also CR3-dependent as myelin failed to phosphorylate FAK in macrophages from CR3 KO mice (Figure 7). These results suggest that myelin induces rapid FAK phosphorylation in CR3-dependent manner.

### PI3K/Akt Serves as Upstream Kinases for NF-κB Activation by Myelin

**Figure 8:**
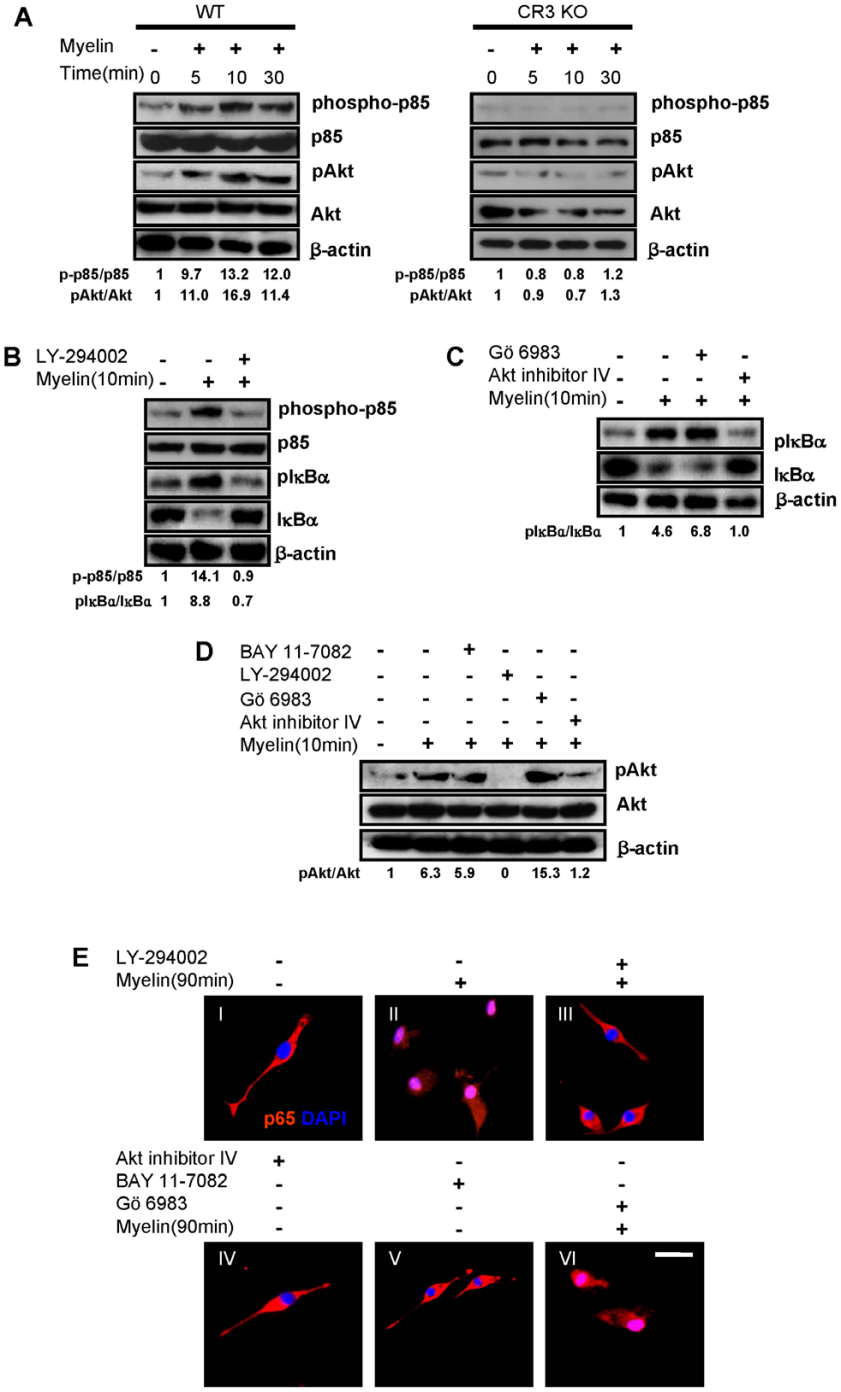
Effect of myelin on activation of PI3K/Akt pathway. (A) Phosphorylation of p85 and Akt in bone marrow-derived macrophages from WT and CR3 KO mice treated with myelin for the indicated time. (B) Effect of PI3K inhibitor LY-294002 on myelin-induced IκB-α phosphorylation and degradation in macrophages. (C) Effect of Akt inhibitor IV on myelin-induced IκB-α phosphorylation and degradation in macrophages. (D) Effect of inhibitors of PI3K, Akt, NF-κB and PKC on myelin-induced Akt phosphorylation in macrophages. (E) Macrophages were pretreated with inhibitors of PI3K, Akt, NF-κB and PKC for 30 minutes, respectively and then incubated with myelin for 90 minutes. Myelin-induced p65 translocation in macrophages was detected by immunofluorescent staining. p65 was stained red and nuclei were stained with DAPI (blue) (Scale bar = 10 µm). All inhibitors were used for 30-minute pre-incubation before myelin incubation. Phosphorylation levels were normalized to non-phosphorylated total proteins. Similar results were obtained from three independent experiments.

**Figure 9:**
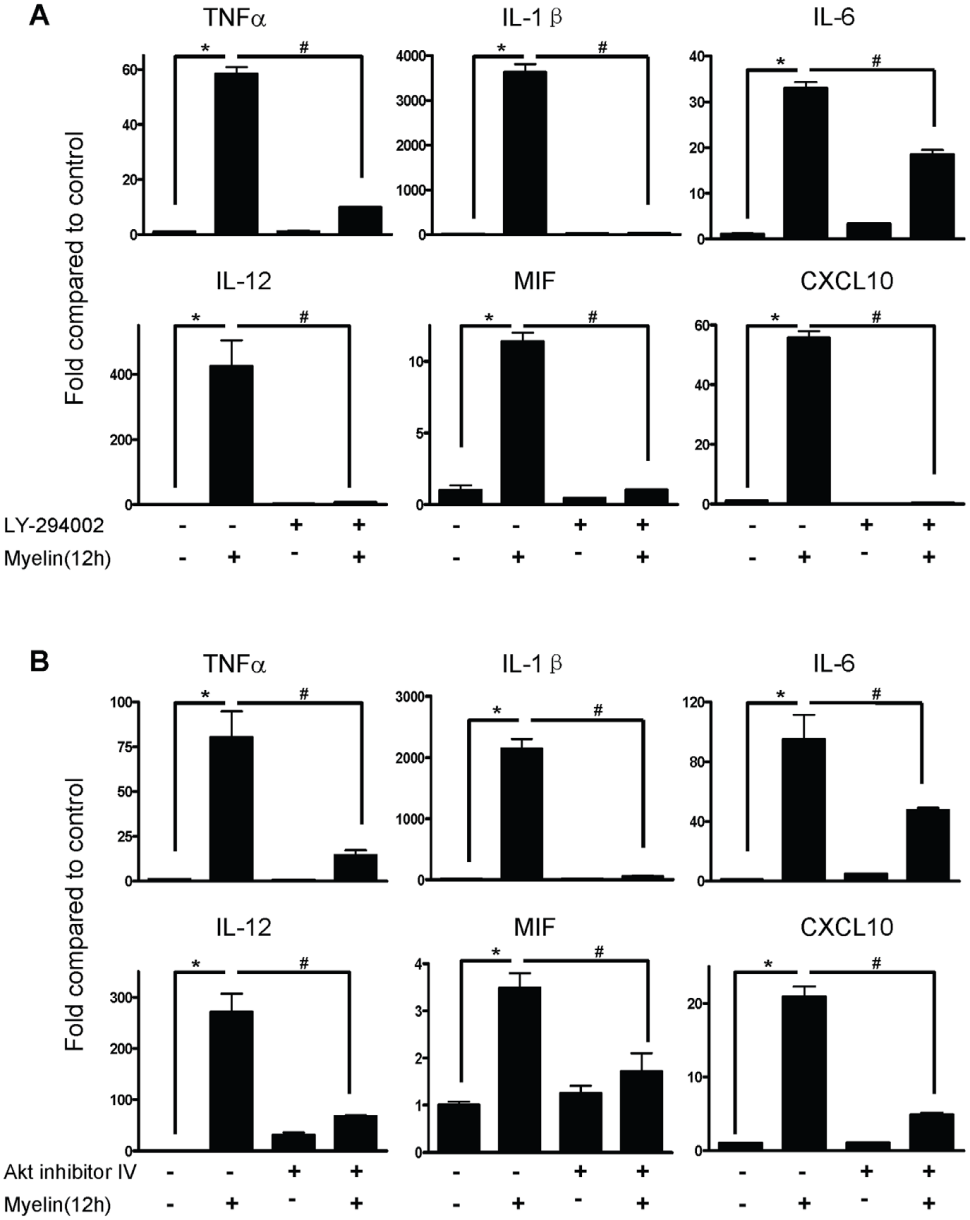
Effect of inactivation of PI3K and Akt on myelin-induced cytokine expression in macrophages. Macrophages were pre-treated with LY-294002 (A) or Akt inhibitor IV (B) for 30 minutes and then treated with myelin for 12 hours. Cytokines at transcriptional level were detected by qRT-PCR. Data were expressed as mean ± SEM and similar results were obtained from three independent experiments. (n = 3, *P<0.05 vs. control; ^#^P<0.05 vs. inhibitor treatment; *t*-test).

FAK can activate CrK/CAS, PI3K and Grb2/Ras cell signaling pathways to regulate cell proliferation, motility, differentiation and intracellular trafficking [16]. As NF-κB can be activated through many different pathways, including phosphatidylinositol 3-kinase (PI3K) and its major downstream kinase, Akt [18], we hypothesize that PI3K/Akt contributes to myelin-induced NF-κB activation. Figure 8A shows myelin treatment phosphorylated p85 regulatory subunit of PI3K and Akt in macrophages from WT mice but not in macrophages from CR3 KO mice. Pre-incubation with PI3K inhibitor, LY-294002 (50 µM) not only effectively inhibited myelin-induced phosphorylation of p85 in macrophages but also inhibited phosphorylation of IκB-α and its degradation induced by myelin (Figure 8B). The Akt inhibitor IV (1 µM), but not protein kinase C (PKC) inhibitor Gö 6983 (5 µM) inhibited IκB-α phosphorylation and degradation in macrophages (Figure 8C). Moreover, phosphorylation of Akt in macrophages can be abolished by both Akt inhibitor IV and PI3K inhibitor LY-294002, but not by PKC inhibitor Gö 6983 and NF-κB inhibitor BAY 11-7082 (Figure 8D), suggesting that Akt is specifically activated by PI3K and Akt is the upsteam molecule of NF-κB. Accordingly, PI3K activating NF-κB was confirmed by immunofluorescent staining of p65 as the inhibitor of PI3K (LY-294002) suppressed p65 translocation from cytoplasm to the nuclei (Figure 8E, I, II and III). Moreover, the Akt inhibitor IV and NF-κB inhibitor BAY 11-7082 readily blocked nuclei translocation of p65 by myelin administration (Figure 8E, IV and V). In contrast, Gö 6983 had no inhibitory effect on p65 nuclei translocation (Figure 8E, VI). These results suggest that PI3K/Akt is upstream kinases for NF-κB signaling and myelin activates the PI3K/Akt/NF-κB signaling pathway. We also showed that inhibitors of PI3K and Akt blocked the myelin induced cytokine production (Figure 9).

## Discussion

Trauma and inflammation damage myelin, creating myelin debris. Myelin debris contains several inhibitors of axonal regeneration [19]. Although the inhibitory effect on axonal regeneration has been well documented, little direct evidence is available concerning myelin-induced changes in inflammation in spinal cord injury. Our findings presented in this study indicated that myelin is a strong inflammatory stimulus that CR3-myelin interaction enhances expression of pro-inflammatory mediators, down-regulates the production of anti-inflammatory cytokines in macrophages, and triggers undesirable inflammation *in vivo*.

These data are crucial for our understanding of the role of myelin in CNS injury and inflammation. Myelin debris generated after CNS injury not only inhibits axonal regeneration and CNS remyelination [20] but also stimulates inflammatory responses. In our present study we showed that the myelin induced inflammatory response is myelin specific as sphingomyelin (the major lipid of myelin) and MBP (one of the major proteins of myelin) are not able to activate NF-κB signaling pathway. Other myelin specific lipids and proteins may have capacity to activate NF-κB. For example, sulfatide, one of major lipid component of myelin sheath, might be a target autoantigen in MS and EAE [21], [22]. Recent *in vitro* results suggested that sulfatide can activate inflammatory response in microglia cells via activation of MAKPs and NF-κB [23]. It is not clear whether CR3 or other receptors is involved in sulfatide-induced NF-κB activation. Further study is needed to investigate whether NF-κB in macrophages can be activated by other myelin specific lipids and proteins.

Myelin not only up-regulates expression of pro-inflammatory mediators such as TNF-α, IL-1β, IL-6, IL-12, MIP-1α and CXCL10 but also down-regulates production of anti-inflammatory cytokines such as IL-4 and TGF-β1. These cytokines play important roles in pathology of CNS injury and inflammation. For example, TNF-α and IL-1β are well-documented pathogenic mediators involved in CNS inflammation and axonal degeneration. Overexpression of IL-6 and IL-12 induced neurodegenerative and inflammatory pathology, respectively [24]. IL-6 and IL-12 deficient mice fail to respond to experimental autoimmune encephalomyelitis (EAE) [25], [26]. IL-4, a well-known anti-inflammatory cytokine, has marked inhibitory effects on the expression and release of the pro-inflammatory cytokines [27], [28]. TGF-β1 exerts potent anti-inflammatory and neuroprotective properties on a LPS-induced model of CNS inflammation and degeneration [29]. CXCL10 and its receptor CXCR3 play a critical role in multiple sclerosis, particularly in leukocyte recruitment into the CNS [30], [31]. Taken together, myelin up-regulation of pro-inflammatory mediators in CNS injury and down-regulation of anti-inflammatory cytokines combine to amply the inflammatory responses.

Kerschensteiner *et al* observed that axonal degeneration and myelin breakdown can happen as early as 30 minutes after injury [32]. In combination with our results, such early myelin debris provides rapid and strong stimuli that stimulate macrophages to produce cytokines including TNF-α and IL-1β and therefore the early increased cytokine expression can be observed after spinal cord injury by Pan *et al* [33] who showed that cytokines mRNA such as TNF-α, IL-6 and IL-1β were increased during the first 2 hours following spinal cord injury, prior to the appearance of circulating cells. Myelin debris is cleared rapidly and efficiently in peripheral nervous system (PNS) [34], [35]. However, myelin debris can persist for years after degeneration of CNS axons as myelin debris can not be efficiently cleared in CNS [36]. Therefore, myelin debris not only provides an early the inflammatory environment for axons but also prolong inflammation, thereby amplifying secondary injury and inhibiting axonal regeneration. Therefore, clearance of myelin debris in CNS may be critical for allowing regeneration.

**Figure 10:**
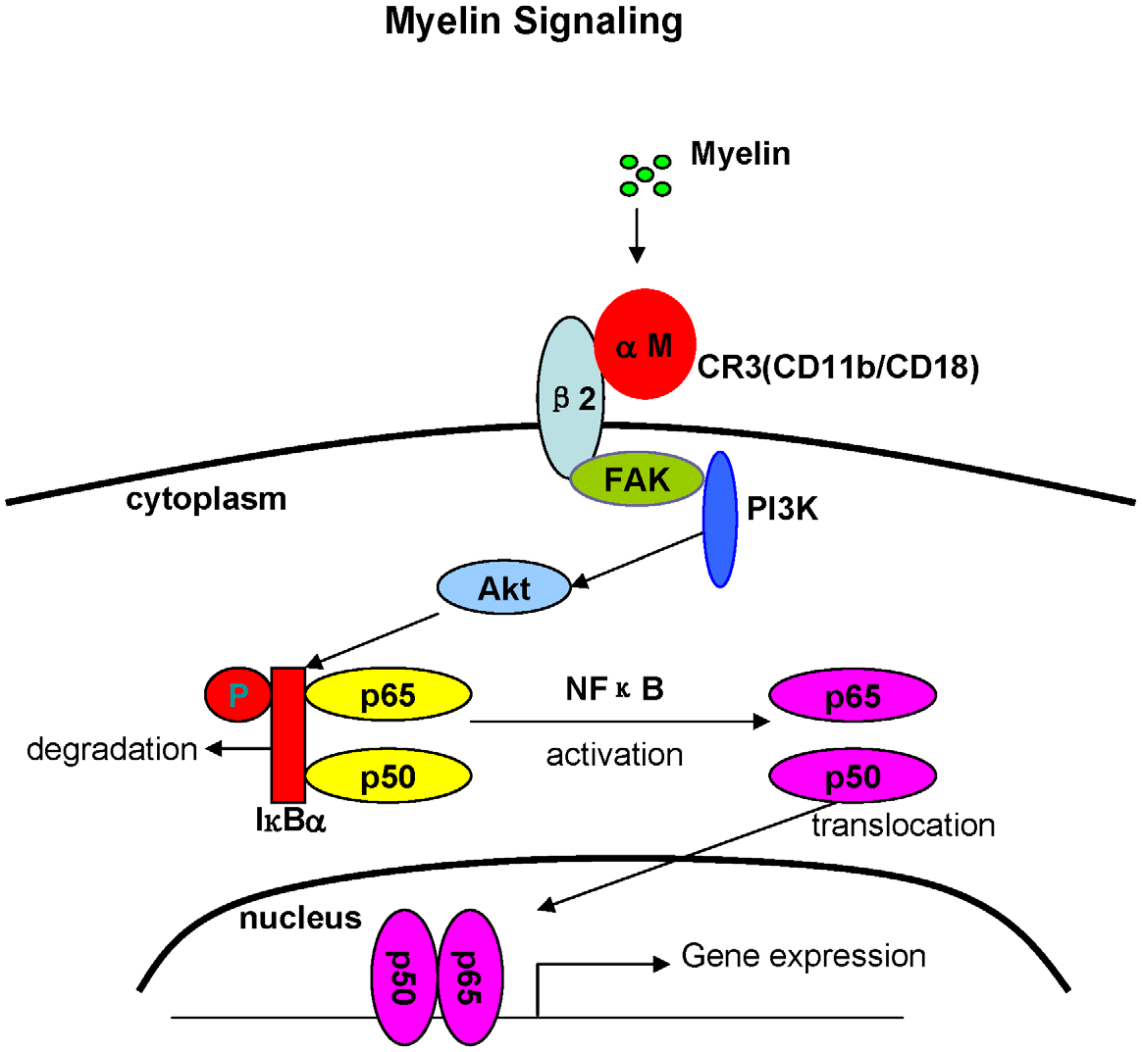
Schematic diagram depicting the possible mechanism that myelin induces inflammatory responses. Myelin debris generated in injured CNS or demyelinating diseases may bind to CR3 and then activate NF-κB through FAK/PI3K/Akt pathway, therefore initiating gene expression of pro-inflammatory mediators.

Myelin can induce macrophages to produce pro-inflammatory cytokines and NO *in vitro* by van der Laan *et al* [13]. However, there were lack of *in vivo* data and cellular mechanism study about how myelin stimulates cytokine production. Understanding the molecular underlying myelin stimulation of inflammatory response should prove vital insights into indentifying new therapies that reduce the secondary damage and enhance regeneration. Our results indicate that myelin strongly activates FAK/PI3K/Akt/NF-κB pathway in macrophages and increases the expression of inflammatory mediators in WT mice but not in CR3 KO mice, suggesting that NF-κB activation by myelin may be CR3-dependent (schematized in Figure 10). Myelin-induced cytokine production is more likely a direct effect as NF-κB can be activated within 5 min, suggesting myelin interacts with macrophages directly rather than the consequence of myelin phagocytosis by macrophages or cytokines released in response to myelin binding to CR3 working in an autocrine manner to trigger NF-κB. Treatment with inhibitors of PI3K, Akt and NF-κB or deletion of CR3 reversed myelin-induced NF-κB activation and also inhibited myelin–mediated production of pro-inflammatory mediators. Therefore, myelin induces cytokine production by activating FAK/PI3K/Akt/NF-κB through CR3-dependent mechanisms. Further studies are needed to identify or rule out other signaling pathways contributing to the myelin-induced cytokine expression.

This study suggests a few strategies to reduce inflammatory response and secondary damage and to promote neuronal regeneration: 1) enhance clearance of myelin debris such as increasing phagocytotic uptake of myelin; 2) inactivation of PI3K/Akt/NF-κB pathway; and 3) targeting CR3-myelin interaction. Although inhibition of NF-κB activity has been shown to reduce inflammation and improves function recovery after SCI [37], enhancement of myelin debris clearance and targeting CR3-myelin interaction after SCI should be further investigated. Leukemia inhibitory factor (LIF) not only acts as a neurotrophic factor [38] in CNS but also inhibits the production of oxygen radicals and TNF-α, and stimulates myelin uptake by macrophages [39], suggesting that LIF may be useful for treating CNS trauma or other demyelinating diseases. In addition, phagocytosis can be also regulated by transmembrane molecules. For example, CD200-CD200R interaction inhibits macrophage/microglia function [40], [41]. Blocking CD200-CD200R interaction and stimulation of macrophage/microglia activation may enhance clearance of CNS myelin debris [36]. Given the crucial roles of CR3 in inflammation and the finding that myelin can initiate inflammatory responses in CR3-dependent manner, blocking CR3-myelin interaction may be beneficial for treatment of CNS injury. It has been shown that myelin clearance is mediated primarily by CR3, however, our *in vitro* and *in vivo* data showed that microglia cells expressing high level of CR3 have poorer efficiency for myelin clearance and myelin clearance by macrophages from CR3 KO mice was not impaired (see Supporting Information Figure S1 and S2), which consistent with the studies from other group showing myelin is not phagocytosed by microglia after SCI [42] and myelin phagocytosis by macrophages may be via galectin-3/Mac-2 [43]. These studies suggested that targeting CR3 may be possible to suppress myelin-induced inflammation without affecting myelin clearance.

## Supporting Information

Table S1 Primer sequences for qRT-PCR
(0.04 MB DOC)

Figure S1 Myelin clearance by macrophages from WT and CR3 KO mice. (A) Bone marrow-derived macrophages from WT and CR3 KO mice were incubated with myelin for 48 hours and cells were stained with Oil Red O and MBP antibody, respectively. (B) In vivo study showed that myelin debris can be phagocytosed by macrophage from WT and CR3 KO mice after spinal cord injury. Representative micrographs of myelin uptake by macrophage in the injured spinal cord at 2 weeks after injury in WT and CR3 KO mice using confocal microscopy. Macrophage was labeled with IBA-1 (green) and myelin debris was stained by Oil Red O (red). Scale bar = 10 µm.
(3.94 MB TIF)

Figure S2 Comparison of myelin uptake by bone marrow-derived macrophages and primary microglia cells. Bone marrow-derived macrophages and primary microglia cells were incubated with myelin for 3 hours. Non-ingested myelin was washed away with PBS and cells were lysed. Intracellular MBP was assessed by ELISA. Data were expressed as mean ± SEM. (n = 3, *P<0.05 vs. control; #P<0.05 vs. microglia cells + Myelin; *t*-test).
(0.00 MB TIF)
