# Supplementary material for: Myelin Activates FAK/Akt/NF-κB Pathways and Provokes CR3-Dependent Inflammatory Response in Murine System
Source: PLoS One. 2010 Feb 23;5(2):e9380. doi: 10.1371/journal.pone.0009380 (PMC2826415; doi:10.1371/journal.pone.0009380)
Supplement: Table S1 — Primer sequences for qRT-PCR (0.04 MB DOC) [file pone.0009380.s001.doc]

**Table S1. Primer sequences for qRT-PCR**

| **cytokine** | **forward** | **reverse** |
| --- | --- | --- |
| **TNF-α** | 5'-ATGCTGGGACAGTGACCTGG-3' | 5'-CCTTGATGGTGGTGCATGAG-3' |
| **IL-1β** | 5'-CCAAAAGATGAAGGGCTGCT-3' | 5'-TCATCAGGACAGCCCAGGTC-3' |
| **IL-6** | 5'-TTCCATCCAGTTGCCTTCTTG-3' | 5'-GAAGGCCGTGGTTGTCACC-3' |
| **IL-12** | 5'-GGATGGAAGAGTCCCCCAAA-3' | 5'-CTGGAAAAAGCCAACCAAGC-3' |
| **MIF** | 5'-CCATGCCTATGTTCATCGTG-3' | 5'-GACTCAAGCGAAGGTGGAAC-3' |
| **MMP9** | 5'-TCGTGGCTCTAAGCCTGACC-3' | 5'-GGTTCAGTTGTGGTGGTGGC-3' |
| **IL-10** | 5'-CCTGGTAGAAGTGATGCCCC-3' | 5'-TCCTTGATTTCTGGGCCATG-3' |
| **IL-4** | 5'-CACGGATGCGACAAAAATCA-3' | 5'-CTCGTTCAAAATGCCGATGA-3' |
| **TGFβ1** | 5'-TGGAGCTGGTGAAACGGAAG-3' | 5'-ACAGGATCTGGCCACGGAT-3' |
| **MIP-1α** | 5'-CCTTGGCTACATTGGTGCAGA-3' | 5'-TGACATGGGCTCCACTGATG-3' |
| **CXCL10** | 5'-GGGCCAGTGAGAATGAGGG-3' | 5'-GCTCGCAGGGATGATTTCAA-3' |
| **MCP-1** | 5'-TTCAGTCTGGTAACATGACGGC-3' | 5'-GGTGCGTTGAGTCTGGCATAA-3' |
