# Supplementary figures and images for: Myelin Activates FAK/Akt/NF-κB Pathways and Provokes CR3-Dependent Inflammatory Response in Murine System
Source: PLoS One. 2010 Feb 23;5(2):e9380. doi: 10.1371/journal.pone.0009380 (PMC2826415; doi:10.1371/journal.pone.0009380)

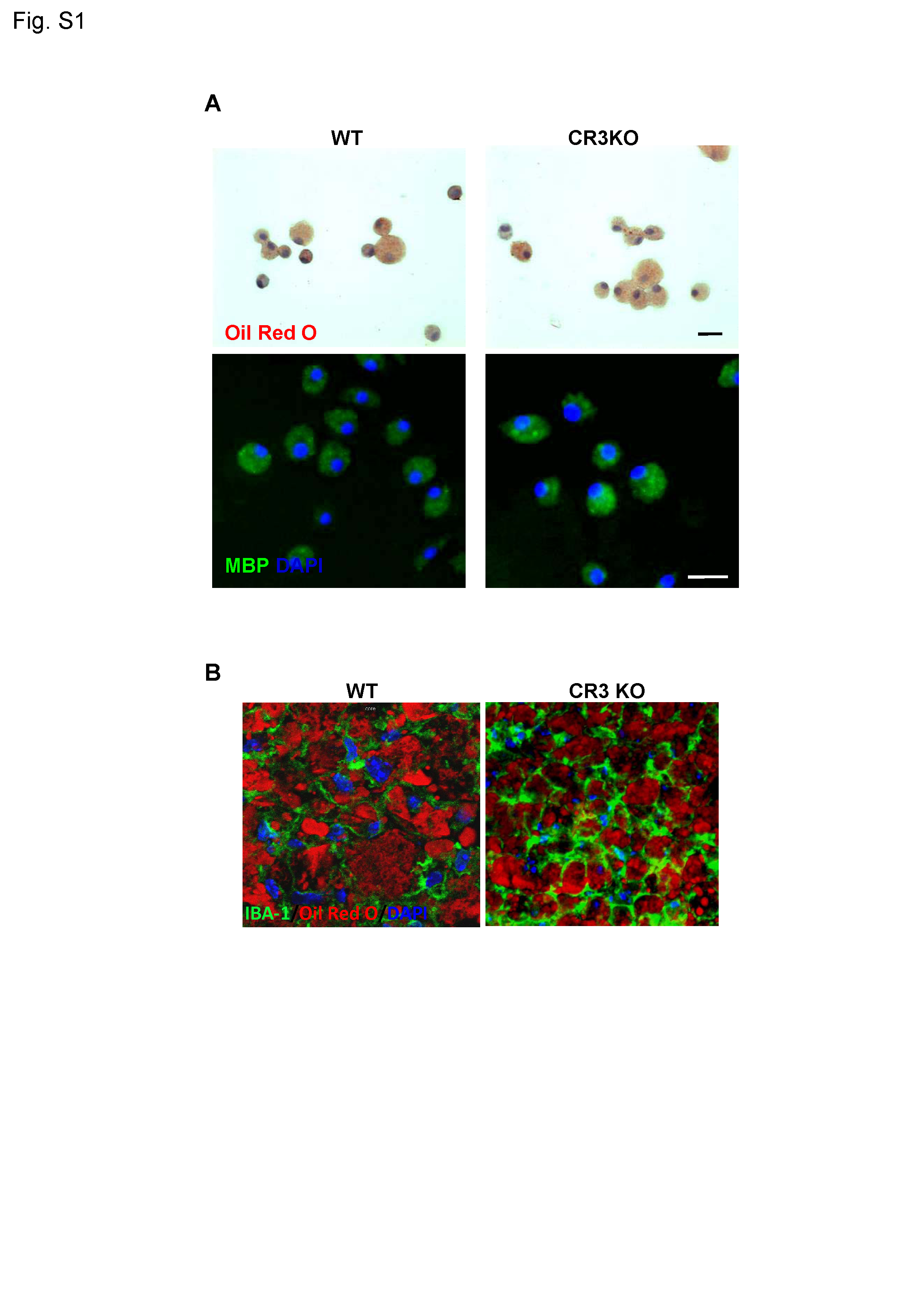

Supplement: Figure S1 — Myelin clearance by macrophages from WT and CR3 KO mice. (A) Bone marrow-derived macrophages from WT and CR3 KO mice were incubated with myelin for 48 hours and cells were stained with Oil Red O and MBP antibody, respectively. (B) In vivo study showed that myelin debris can be phagocytosed by macrophage from WT and CR3 KO mice after spinal cord injury. Representative micrographs of myelin uptake by macrophage in the injured spinal cord at 2 weeks after injury in WT and CR3 KO mice using confocal microscopy. Macrophage was labeled with IBA-1 (green) and myelin debris was stained by Oil Red O (red). Scale bar = 10 µm. (3.94 MB TIF) [file pone.0009380.s002.tif]
